# Colorful and facile in situ nanosilver coating on sisal/cotton interwoven fabrics mediated from European larch heartwood

**DOI:** 10.1038/s41598-021-01914-y

**Published:** 2021-11-17

**Authors:** K. M. Faridul Hasan, Péter György Horváth, Zsófia Kóczán, Miklós Bak, Tibor Alpár

**Affiliations:** 1 Simonyi Károly Faculty of Engineering, University of Sopron, Sopron, Hungary; 2 Paper Research Institute, Simonyi Károly Faculty of Engineering, University of Sopron, Sopron, Hungary

**Keywords:** Nanoparticles, Synthesis and processing, Nanobiotechnology, Nanotoxicology

## Abstract

This study reports on a novel coloration approach for sisal/cotton interwoven fabric via in situ synthesis of European larch (*Larix decidua*) heartwood-anchored sustainable nanosilver. The heartwood extracts functioned as the reducing and stabilizing agent in reaction systems. The deposited silver nanoparticles (AgNPs) over the fabric surfaces displayed brilliant coloration effects with improved fastness ratings and color strengths (K/S). The successful depositions of nanosilvers were quantified and increasing trends in K/S values with the increase in silver precursor loading were discovered. The concentrations of AgNPs deposited on fabric surfaces were found to be 16 mg/L, 323 mg/L, and 697 mg/L, which were measured through an iCP OES (atomic absorption spectroscopy) test. The K/S values obtained for different loadings of silver precursors (0.5, 1.5, and 2.5 mM (w/v)) are 2.74, 6.76, and 8.96. Morphological studies of the control and AgNP-treated fabrics also displayed a uniform and homogeneous distribution of AgNPs over the fabric surfaces. FTIR (Fourier transform infrared spectroscopy) studies of the sustainably developed materials further confirms the successful bonding between the fabrics and AgNPs. Furthermore, stability against temperature was also noticed as per TGA (thermogravimetric analysis) and DTG (derivative TG) analysis although there was a slight decline from the control sisal/cotton interwoven fabrics observed. Statistically, regression analysis and ANOVA tests were conducted to understand the significance of increased nanosilver loading on sisal/cotton interwoven fabrics. In summary, the perceived results demonstrated successful coloration and functionalization of sisal/cotton interwoven fabrics through green AgNPs, which could indicate a new milestone for industrial production units.

## Introduction

The functionalization of natural fibers as well as associated fabrics with various nanoparticles has garnered significant attention in both daily life and in industrial production. Among different nanoparticles^1,2^, AgNPs is the most prominent and popular due to its optical, coloration, bacterial resistance, and thermal performances^3–5^. AgNPs display scattering and light absorption properties, especially in their oscillation of free electrons in the presence of incident light, which is termed LSPR (localized surface plasmon resonance). Metallic nanoparticles expose diversified colors depending on the LSPR wavelength and nanoparticles size, shape, and dielectric and inter particular distance^6^. Even though the existence of AgNPs were not confirmed in ancient times, the technology was still employed in the stained glass windows of Western churches and in lycrogus cup colorations^7,8^. However, the coloration of different natural and synthetic fibers using biosynthesized AgNP is still a novel approach. Research focusing on the coloration and functionalization of natural fibers derived substrates and associated fabrics has been extensive^9–12^. Some studies have emphasized the necessity of cationization of natural fibers like cotton^13^. Nevertheless, any extra processing requires additional chemicals, associated energy, and operational set up, which incurs additional production costs. Though coloration of blended fiber and interwoven fabric-based textile with advanced technologies possesses significant application potential for industrial production and consumers, research into these methods has been limited. Consequently, interwoven sisal/cotton woven fabric coloration generally occurs without pretreatments under the justification that this omission saves on utility costs during operations.

Sisal is a potential source of naturally derived fabric and a prospective source of multifunctional textiles. Recent studies have utilized and functionalized sisal to develop composite materials^14,15^. However, the coloration/functionalizing of sisal using nanosilver-based materials has not been studied yet. Furthermore, several attempts to functionalize sisal fibers using different types of nanoparticles have been made^16,17^. On the other hand, cotton-due to its superior hygroscopicity, heat retention, and softness-is the dominant natural fiber in textile industries^18^. As noted in Table 1, cotton consists mainly of cellulose, lignin, hemicellulose, and oil and wax. Sisal fibers display chemical characteristics that are similar to cotton because both cotton and sisal are naturally originated fibers. A great deal of research has been conducted on cotton, as it is the most widely used material in clothing. Researchers also attempted to functionalize cotton with a various nanoparticles such as ZnO, Cu, TiO_2_, and SiO_2_^19–21^. However, researchers have also focused considerable efforts on AgNP to color and functionalize cotton as well^13,22,23^. Recently, researchers have also concentrated on the green synthesis of AgNPs using different naturally derived materials such as plants, flowers, stems, and leaves^10,24–26^. Although a variety of plants that could be used to synthesize AgNPs has been investigated, the heartwoods of European larch as a stabilizing and reducing agent of silver precursors have not been studied yet. European larch is widely grown in central European countries like Hungary. Due to its colorful and aesthetic texture, European larch wood is extensively used in various outdoor applications including rails, terraces, windows, and fences as well as in indoor applications such as furniture, flooring, and stairways^27^. However, the heartwoods extracts of European larch can also be utilized as a reducing and stabilizing agent. The present study aims to demonstrate the immense possibilities of European larch in coloration.

**Table 1:** Chemical properties of sisal and cotton.

Fibers	Sisal^29^ (wt.%)	Cotton^29,30^ (wt.%)
Cellulose	65	85‒90
Hemicellulose	12	4‒5.9
Lignin	9.9	0.75
Oil and wax	2	0.6

Technological advancements have made comfort enhancement a driving force in fabric design. In earlier fabric production, fabrics were produced wholly from the same category of fibers, like 100% cotton or 100% polyester, for example. Later, different proportions of fibers were blended together to produce more flexible fabric blends from raw materials like 65% cotton and 35% polyester. Scientists eventually utilized production technology to produce interwoven fabrics from different categories of fibers, such as yarn to produce woven fabrics. In order to produce interwoven fabrics, it is necessary to insert warp and weft yarns of different fibers in the same fabric structure. In woven structured fabrics, lengthwise yarns are termed “warp”, while crosswise yarns are called “weft”^28^. Therefore, interwoven engineered fabrics made of warp (sisal fibers) and weft (cotton fibers) were selected for this study. The coloration of sisal/cotton interwoven fabric is also an important wet-based technology, although dyeing is still performed using traditional dyeing protocols and technology using reactive dyes, which require extensive chemicals and dyestuffs. The usage of traditional colorants poses constant threats to the environment. As a result, there is an urgent demand to explore feasible and advanced technological processes to color interwoven cellulosic fabrics in an eco-friendly way. Moreover, interwoven fabrics made of sisal and cotton fibers for nanosilver-based coloration and functionalization has not yet been explored. The current study presents a novel method for the coloration of sisal/cotton interwoven fabrics using European larch heartwood-mediated AgNPs. This study offers the interwoven coloration industry new operational technology and sustainable coloration possibilities with significant potentiality.

## Experimental

### Materials

Grey-colored sisal/cotton interwoven fabrics of 220 GSM (g/m^2^) density with a structure of interwoven fabric with cellulosic paper mat underneath was procured from Locatelli Hungária Kft. (Hungary). Initially, fabric parts were separated from the mat via boiling at 90 °C for 15 min. In the sisal/cotton interwoven fabrics, sisal yarns were engineered in warp direction, whereas cotton yarns were in weft direction. The sisal/cotton fabric samples were then washed and dried at 100 °C for 12 min. Later, 5 g samples were measured for every single nanosilver treatment. In order to synthesize AgNP, AgNO_3_ reagent was procured from Sigma Aldrich Co., St. Louis, United States. European larch heartwood (scientific name *Larix decidua*) was collected as wood logs from the University of Sopron woodworking shop.

### In situ biosynthesis of green AgNPs on sisal/cotton interwoven fabrics

**Figure 1:**
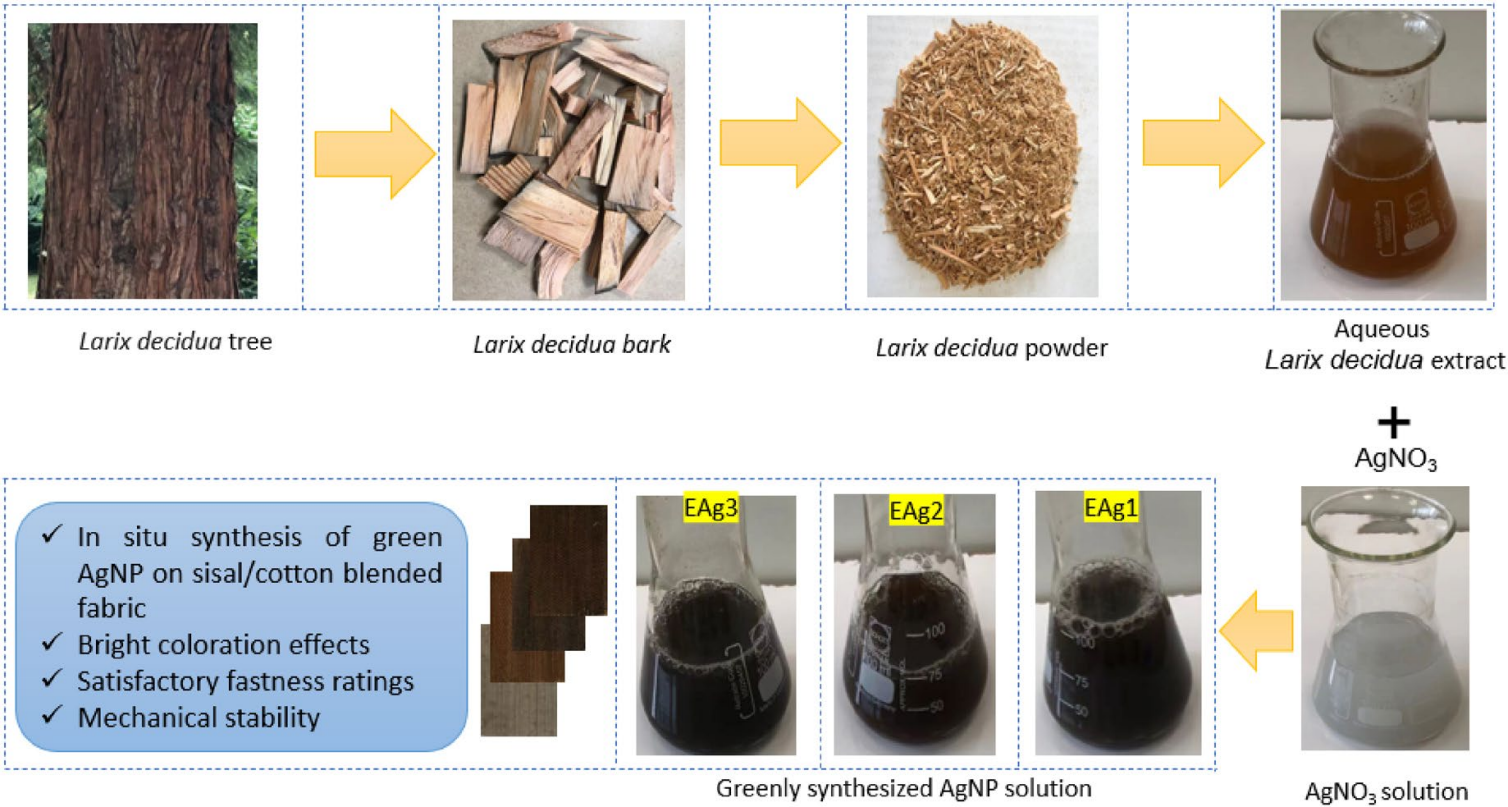
A schematic operational protocol for the extraction of European larch bark aqueous solution and in situ biosynthesis of nanocolloids on sisal/cotton interwoven fabrics.

**Figure 2:**
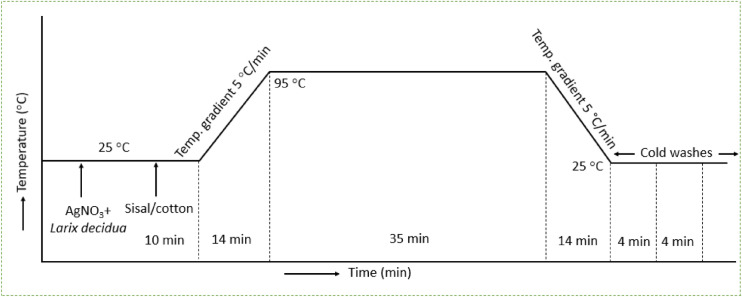
Operational procedures of green AgNPs deposition mechanism on sisal/cotton interwoven fabrics.

The heartwood logs of European larch were directly collected from the woodworking shops at the University of Sopron, Hungary. The collected heartwood logs were cut into smaller pieces with a laboratory circular saw machine (DCS570N XJ model, Pennsylvania, United States)). The woods pieces (Fig. 1) were then washed with distilled water to remove any unexpected debris and dust. This was followed by drying at 120 °C for 15 min. The heartwood pieces were then crushed with a laboratory grinder until they attained powder form to ensure easy extractions into aqueous solutions. Around 100 g of *Larix decidua* powder was mixed with 500 mL of water to secure a 20% w/v aqueous solution at boiling temperature around 100 °C for 30 min. The aqueous solution was then filtered and wood wastes were removed. Afterward, the sealed and bottled extracted solution was stored in a freezer at around 4 °C for later use. AgNO_3_ and *Larix decidua* solutions of different concentrations, as tabulated in Table 2, were measured and mixed in the beaker. The beaker was then continuously stirred until an optimal solution was prepared. The first sample, in which 0.5 mM AgNO_3_ was used, was denoted as EAg1. The second (EAg2) and third sample preparation (EAg3) used 1.5 and 2.5 mM AgNO_3_, respectively. The aqueous solutions of *Larix decidua* were used in 4.0 MW in % (v/v) in all cases. Both AgNO_3_ and *Larix decidua* aqueous solutions were then mixed properly with a magnetic stirrer. The sisal/cotton interwoven fabrics were then immersed in the solutions and stirred for another 10 min. The color of the solutions started to change from a milky appearance to a colored state reflecting the development of a nanosilver environment in the beaker. The successful attachment of the colorful nanosilver on fabric surfaces were performed at 95 °C for 35 min. Figure 2 illustrates the detailed operational mechanism. Finally, the beakers were cooled and the samples were washed with distilled water three times at room temperature (25 °C) to remove any poorly adhered nano seeds from fabric surfaces. The samples were then subsequently dried in a laboratory dryer at 120 °C for 12 min.

**Table 2:** Recipe protocols for control and nanosilver-coated sisal/cotton interwoven fabrics.

Specimens	AgNO_3_ concentrations (mM)	EL (MW in % (w/v))
EAg0	0	0
EAg1	0.5	4.0
EAg2	1.5	4.0
EAg3	2.5	4.0

*EL* European larch heartwood, *MW* molar weight.

### Characterizations of deposited AgNPs on sisal/cotton interwoven fabrics

The moisture contents of the heartwoods and nanotreated fabrics were investigated using a moisture analyzer (Kern ULB 50‒3 N model instruments, KERN AND SOHN GmbH company, Germany). Initially, the Ag contents on aqueous solutions were measured using atomic absorption spectroscopy, iCP OES, iCAP™ 6300 series, Thermo scientific, USA. The morphological observations of the control and AgNP-treated fabrics were conducted using an SEM (scanning electron microscopy) machine (SEM, S 3400 N Hitachi model, manufactured in Tokyo, Japan) at 8.00 kV. Moreover, special software (Quantax Esprit 1.9, Berlin, Germany) was used to assess the elemental compositions (EDX) and mapping. The perceived color values were measured using Konica Minolta spectrophotometer (model: CM-2600d, Japan) within 400 to 700 nm wavelengths ranged under D65 observer (standard). The chemical bonding structures of control and nanosilver treated materials were tested in terms of FTIR spectroscopy analysis within 4000 to 400 cm^‒1^ wavenumber. Thermal properties were evaluated with a Themys thermal analyzer, Setaram Instrumentation, France in terms of TGA and DTG under nitrogen atmosphere at 10 °C/min temperature gradient ranged from 10 to 800 °C. The fastness properties of the colored products were also investigated further; whereas wash fatness properties were assessed using ISO 105C06:1994 standard and rubbing fastness properties by ISO 105‒X12:2003 methods by a crock meter. Standard laboratory environment was considered for the testing (65 ± 5% relative humidity and 20 ± 5 °C) for temperatures. Finally, grey scale was implemented to assess the ratings according to the respective tests in terms of shade changes in the clothes.

### Ethical approval

This research study involved no human participants or any animal experiments. The European larch (*Larix decidua*) heartwood used in this research is complied with local institutional, national, and international guidelines and legislation. The necessary permissions for the used plant is also taken from respective authorities.

## Results and discussions

### In situ synthesis mechanism of AgNPs on sisal/cotton interwoven fabrics

Nanosilvers were in situ synthesized over sisal/cotton interwoven fabrics by utilizing *Larix decidua* aqueous solutions as the green capping and reducing agent for AgNO_3_. When the *Larix decidua* aqueous solutions (4.0%, v/v) were dropped in the beakers containing AgNO_3_, the color of the solutions started to change visually from a milky state to a yellowish brown. After several minutes of mixing, the color turned completely dark brown, demonstrating a successful formation of metallic AgNPs. The Ag^+^ is reduced to metallic (Ag^0^) after a good mixing of a silver precursor and *Larix decidua* aqueous solution, especially for the photocatalytic actions^31^. Another researcher presented a similar report^32^. Moreover, the color changes also occurred for the oscillation of conduction electrons in metallic nanoparticles due to LSPR properties of AgNPs. Furthermore, the color intensity depends on increased concentrations of used silver precursor in the synthesis system.

### Quantification of control and AgNP treated sisal/cotton interwoven fabrics

**Figure 3:**
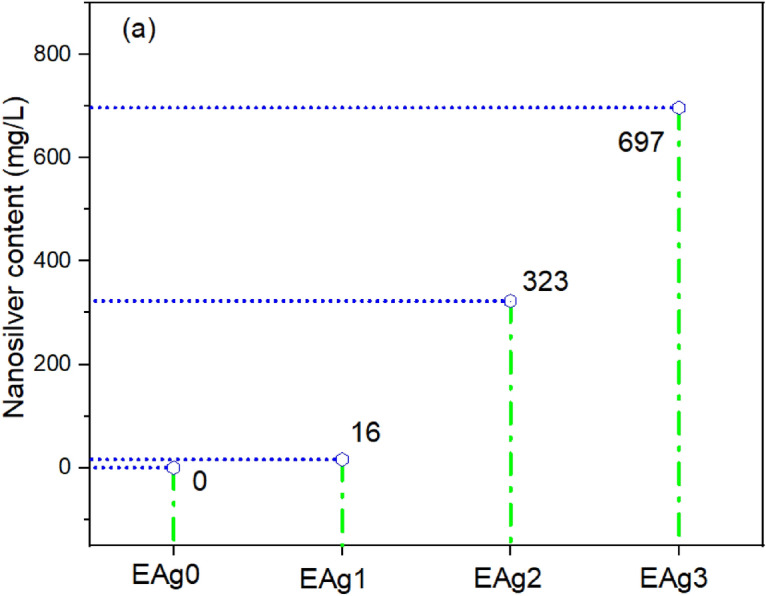
iCP OES test analysis for nanocolloids developed over sisal/cotton interwoven fabrics.

The nanosilvers present on aqueous solutions were investigated with an iCP OES test. AgNPs present on nanocolloid for 0.5, 1.5, and 2.5 mM AgNO_3_ (Table 2) precursors were 16, 323, and 697 mg/L, respectively (Fig. 3). Furthermore, AgNP contents increased with the increase of AgNO_3_ in the nanocolloid environments. Similar findings were also reported in our recently published works on nanosilvers mediated from *Fraxinus excelsior* tree flowers^4^. Therefore, it could be concluded that the presence of AgNPs could be tuned and regulated by controlling the silver precursor in the reaction systems.

### Morphological, EDX spectra, and elemental observations of control and AgNPs deposited on sisal/cotton interwoven fabrics

**Figure 4:**
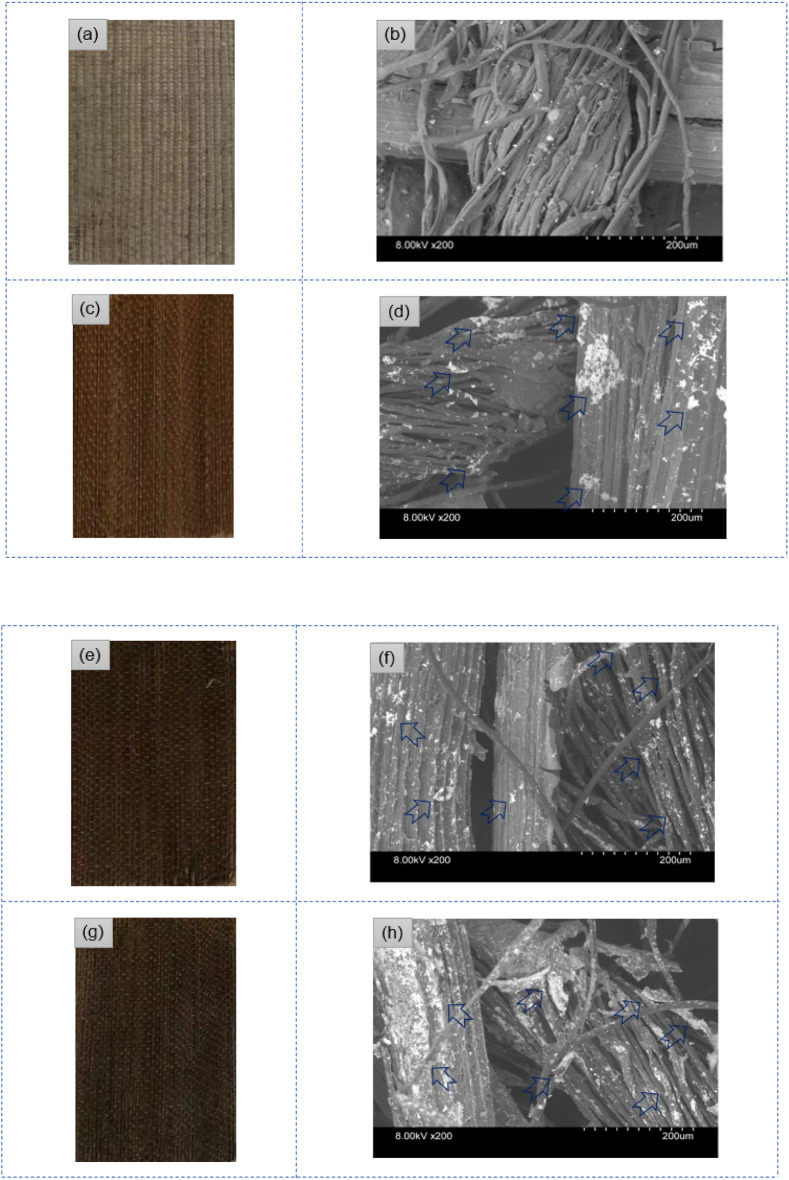
Physical photographs of control and nanosilver treated samples: (**a**) control sisal/cottons physical image, (**b**) control sisal/cottons morphological image, (**c**) physical image of EAg1, (**d**) morphological image of EAg1, (**e**) physical image of EAg2, (**f**) morphological image of EAg2, (**g**) physical image of EAg3, (**h**) morphological image of EAg3.

The morphological observations clearly demonstrated the presence of AgNPs on sisal/cotton interwoven fabrics (Fig. 4). A strong binding was created between the nanosilver and fabrics, which enabled the excellent interactions between them. All the fabric samples heavily displayed the presence of AgNPs, whereas control samples did not show any AgNP presence, only plain and uniform surfaces. The coated and deposited nanoparticles were also evident at the surfaces and inter-fiber spaces of the fabrics. Furthermore, the nanotreated samples also displayed a size and shape that is typical of the metallic AgNP^33^.

**Figure 5:**
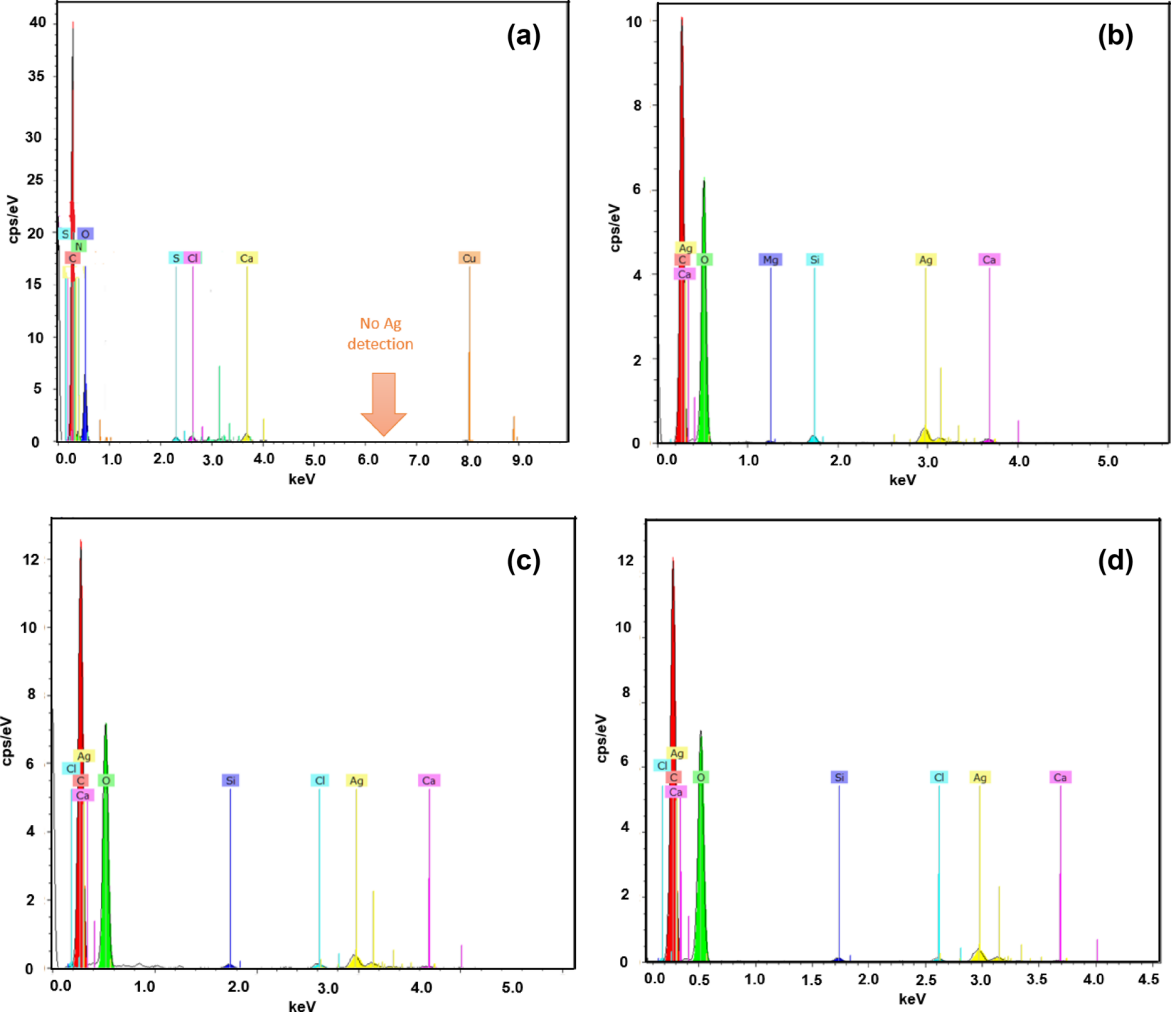
EDX spectra of the control and nanosilver treated sisal/cotton interwoven fabric specimens: (**a**) control sisal/cotton, (**b**) EAg1, (**c**) EAg2, and (**d**) EAg3.

**Figure 6:**
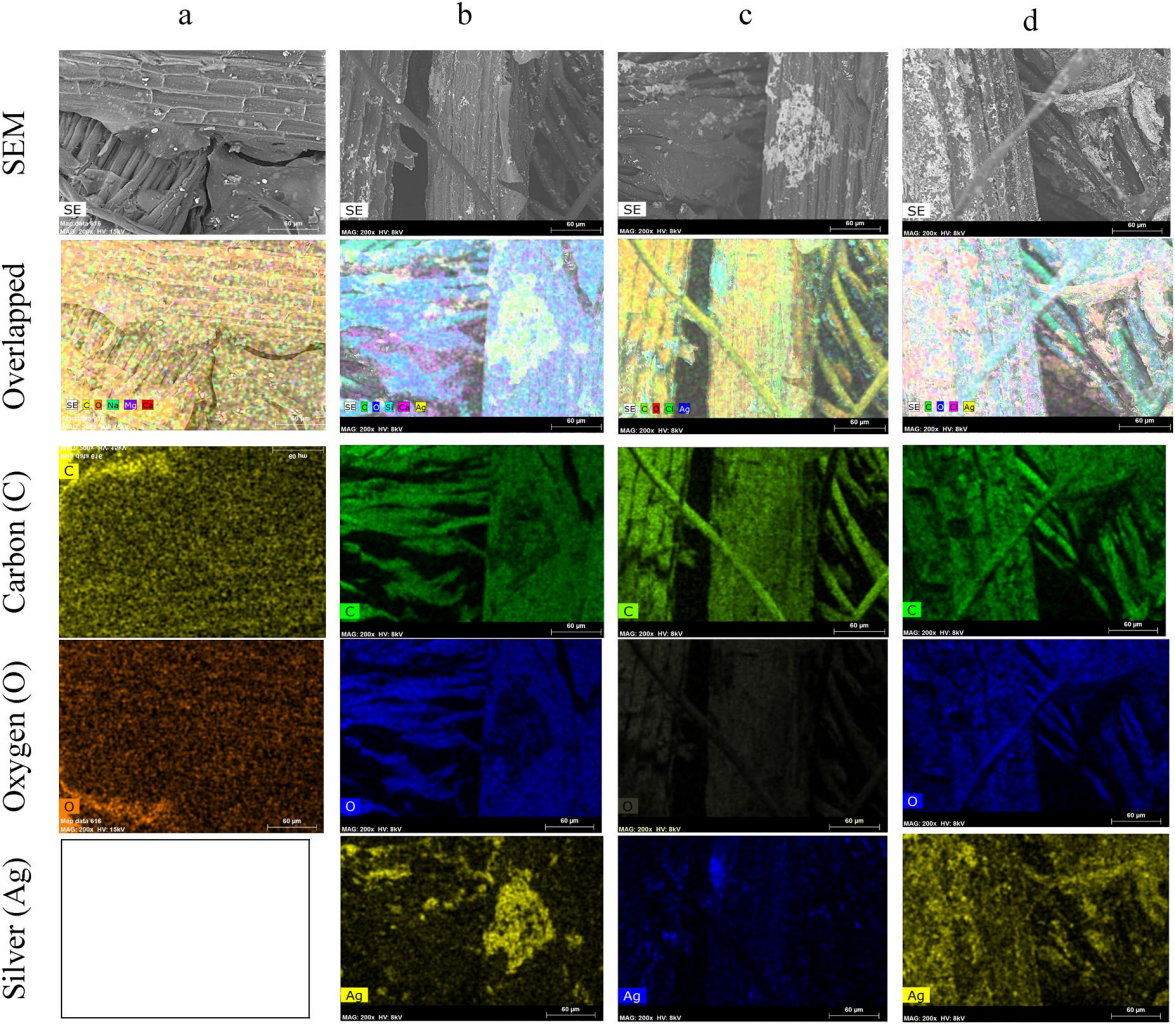
Elemental mapping images of control and AgNP treated sisal/cotton interwoven fabric specimens: EAg0 (**a**), EAg1 (**b**), and EAg2 (**c**), and (**d**) EAg3.

The presence of metallic AgNPs was also confirmed by SEM-deployed EDX analysis (Fig. 5). All the nanoparticle coated samples exhibited a typical peak at around 2.96 keV, which was absent from the control sample^34^. Moreover, in addition to the presence of C and O (typical chemical elements of cellulosic materials and metabolites present in plants) in all the samples, Cl was also present. This may have been due to the presence of plant-derived materials. In addition, all plants inherently possess ample nutrients like chloride ions^34,35^. Other impurities like S, Si, and Ca were observed in small amounts, attesting to the possibility that these may have been generated during processing. The wt% of C and O did not vary significantly within the nanosilver treated fabrics but it did vary within the control (50.08 and 47.94%, respectively) and other samples (41.37, 45.6, and 43.39% C and 44.1, 43.3, and 43.1% O, respectively for samples 2, 3, and 4). Furthermore, there was a slight difference in wt% between 0.5 and 1.5 mM silver precursor treated samples where the values were 9.462% and 9.463%, respectively (Table 3). However, the wt% of sample 4 revealed significant differences in nanosilver contents (9.95%), which was around 5.15% higher than in samples 2 and 3. A similar detection of chemical elements (C and O for the control sample and Ag for the nanocoated samples) in the control and nanosilver treated fabrics was also observed in elemental mapping analysis. A homogeneous and uniform distribution of C, O, and Ag was also explicitly observed in the overlapping images (Fig. 6) demonstrating successful reinforcement effects between the metallic silver and cellulosic fabric substrates. The discussions further confirmed the successful binding and deposition of green nanosilver on sisal/cotton interwoven fabrics.

**Table 3:** Numerical values of EDX analysis for control and nanosilver-coated sisal/cotton interwoven fabrics.

Samples	Carbon (wt.%)	Oxygen (wt.%)	AgNP (wt.%)	Others (wt.%)
EAg0	50.08	47.94	–	< 2.5
EAg1	41.37	45.6	9.462	< 4
EAg2	43.39	44.1	9.463	< 3.5
EAg3	43.3	43.1	9.95	< 4

### Color and fastness studies of control and AgNP treated sisal/cotton interwoven fabrics

Color characteristics of control and nanosilver-coated sisal/cotton interwoven fabrics were also measured in order to investigate the effects of AgNPs on grey fabrics. Although the changes of color appearances in nanocolloids were observed visually, the colorful solid samples (nanosilver deposited) were assessed with the widely used spectrophotometric data, which is also considered a simple method to apply to colorimetric study. The investigated colorimetric data include K/S values and color coordinates (L*, a*, and b*) as shown in Table 4. Therefore, the variation in color sensing capabilities were determined in terms of K/S and CIE (L*, a*, and b*) values for all the test specimens. Table 4 clearly demonstrates that coloration using different treatments over sisal/cotton interwoven fabrics employing AgNO_3_ has significant effects on perceived colors. The control fabric samples exhibited nearly white and lighter color tones (84.02) with very low K/S value (0.09) and with comparatively lower a* (1.89) and b* (6.8) values as well. The K/S values started to increase with the increase of AgNPs in the test samples. Test specimen 2 possessed only 2.74 K/S values, whereas sample 3 showed 6.76 and sample 4, a value of 8.96. In addition, it is further noticed that K/S values shown by EAg1 is 30.44, EAg2 by 75.11, and EAg3 by 100 times higher compared to control sample, which clearly confirmed that AgNP concentrations played a vital role regarding this changes.. This clearly confirmed that AgNP concentrations played a vital role in these changes. Conversely, L* values indicating the darkness/lightness values also exhibited a decreasing trend. The increase of silver contents by sample were as follows: from 84.02 for the control to 42.46, 29.74, and 25.95 for samples 2, 3, and 4, respectively. The Ag contents for the three samples were 16, 323, and 697 mg/L, respectively. As was the case with K/S values, increased Ag loading also made fabrics darker by as much 1.5, 1.7, and 1.87 times. In addition, the other values like a* and b* also showed positive integers, indicating the presence of reddish/yellowish tones in the color coordinates. Therefore, the colors displayed ranged from light orange (sample 2), to orange red (sample 3), and dark orange red (sample 4). Another reason for the color darkness was the increase of silver content formed more Ag^0^; hence, the colors became darker due to LSPR effects. Finally, successful formations of colorful AgNPs were found on the surfaces of sisal/cotton interwoven fabrics. The perceived results achieved and discussed here are in agreement with other reports^33,36^.

**Table 4:** Colorimetric data for control and nanosilver-coated sisal/cotton interwoven fabrics.

Samples	K/S	L*	a*	b*	Color evaluation
EAg0	0.09	84.02	1.89	6.8	Grey/nearly white
EAg1	2.74	42.46	12.98	38.64	Light orange
EAg2	6.76	29.74	16.84	47.56	Orange red
EAg3	8.96	25.95	17.19	43.19	Dark orange red
Coefficient of variations (R^2^)	0.95	0.99	0.99	0.99	–

### Statistical analysis of color characteristics

Initially, regression analysis for the coloration properties of all the samples were carried out in terms of nanosilver loading on sisal/cotton interwoven fabrics. Besides, the R^2^ values as shown in Table 4 found for all the samples equal or higher than 0.95 demonstrating that the higher loading of AgNPs on fabric samples exist a positive attribution in terms of K/S, L*, a*, and b* values. Furthermore, the perceived values in case of p are found to have less than 0.05 for all the coloration characteristics just except for the intercept in K/S value (marked through bold color), also indicate that there are significant effects of increased nanosilver loading on fabrics (Table 5, Table 6, Table 7, Table 8). Moreover, significance of the results was also further investigated by means of one-way ANOVA test where the samples were considered as categorical factor. The associated F-tests also demonstrate the significance of all the coloration values. Moreover, pairwise comparison of different samples were also conducted by Fisher LSD test, reflecting a significant differences within all the coloration values as the p value is less than 0.05 (assumed level) (Table 9, Table 10, Table 11, Table 12). However, a* value did not provide significant differences in two cases (Table 11) as marked through bold color. The similar effects of statistical analysis was reported in our previous study on flax/glass reinforced methylene diphenyl diisocyanate composite materials to understand the effects of glass in the system^37^. Otherwise, the way we presented the statistical data in this current study is not available by other similar studies for understanding the influences of AgNPs in sisal/cotton interwoven fabrics.

**Table 5:** Regression analysis for K/S values in terms of nanosilver loading.

Effects	Parameter	Standard error	T	*P*
Intercept	0.600733	0.399822	1.50250	**0.163871**
Composition	0.163562	0.012322	13.27343	0.000000

Bold *p* values indicate not significant.

**Table 6:** Regression analysis for L* values in terms of nanosilver loading.

Effects	Parameter	Standard error	t	*P*
Intercept	68.64611	6.139479	11.18110	0.000001
Composition	-0.92900	0.189218	− 4.90968	0.000614

**Table 7:** Regression analysis for a* values in terms of nanosilver loading.

Effects	Parameter	Standard error	t	*P*
Intercept	6.210323	1.794346	3.461050	0.006112
Composition	0.247529	0.055302	4.475982	0.001186

**Table 8:** Regression analysis for b* values in terms of nanosilver loading.

Effects	Parameter	Standard error	t	*P*
Intercept	19.56498	5.305691	3.687546	0.004194
Composition	0.57852	0.163521	3.537891	0.005376

**Table 9:** Fisher LSD test for K/S values in terms of different samples (nanosilver loading).

Samples	1	2	3	4
EAg0		0.003878	0.000011	0.000001
EAg1	0.003878		0.000507	0.000014
EAg2	0.000011	0.000507		0.005544
EAg3	0.000001	0.000014	0.005544

**Table 10:** Fisher LSD test for L* values in terms of different samples (nanosilver loading).

Samples	1	2	3	4
EAg0		0.000000	0.000000	0.000000
EAg1	0.000000		0.000002	0.000000
EAg2	0.000000	0.000002		0.002788
EAg3	0.000000	0.000000	0.002788

**Table 11:** Fisher LSD test for a* values in terms of different samples (nanosilver loading).

Samples	1	2	3	4
EAg0		0.000000	0.000000	0.000000
EAg1	0.000000		0.000001	0.000002
EAg2	0.000000	0.000001		**0.206463**
EAg3	0.000000	0.000002	**0.206463**	

Bold *p* values indicate not significant.

**Table 12:** Fisher LSD test for b* values in terms of different samples (nanosilver loading).

Samples	1	2	3	4
EAg0		0.000000	0.000000	0.000000
EAg1	0.000000		0.000064	0.006111
EAg2	0.000000	0.000064		0.004621
EAg3	0.000000	0.006111	0.004621	

The color characteristics are not restricted only to imparting color on fabrics, but also resist fading under certain conditions such as exposure to water and rubbing. Therefore, the durability of colors deposited on sisal/cotton fabrics was investigated in terms of washing and rubbing (both dry and wet conditions). The color durability of Sample 2 (lowest silver-containing fabric) was better with a higher loading of nanosilver. However, all the samples displayed better color stability. Sample 2, with a wash fastness value of 4, demonstrated “very good” color stability, whereas samples 3 and 4 had a rating of 3, indicating “good” color fastness to washing. On the other hand, samples 2 and 3 displayed a value of 3 for wet rubbing, which reflects “good” color durability. Sample 4 exhibited a value of 2, indicating “fair” fastness properties (Table 13). The trend in dry rubbing was nearly the same as the wet rubbing trend, which demonstrates “good” fastness characteristics, provides detailed data concerning the fastness of colorful nanosilver-coated sisal/cotton interwoven fabrics. The phenemenon found above also goes in agreement with some previous studies for AgNP colored textile substrates (natural fiber-based)^38,39^.

**Table 13:** Fastness characteristics for control and nanosilver-coated sisal/cotton interwoven fabrics.

Samples	Wash fastness	Description	Wet rubbing	Description	Dry rubbing	Description
EAg0	‒		‒		‒	
EAg1	4	Very good	3	Moderate	3	Moderate
EAg2	3	Moderate	3	Moderate	2‒3	Slightly fair
EAg3	3	Moderate	2	Fair	2	Fair

### FTIR investigations of control and nanosilver-coated sisal/cotton interwoven fabrics

**Figure 7:**
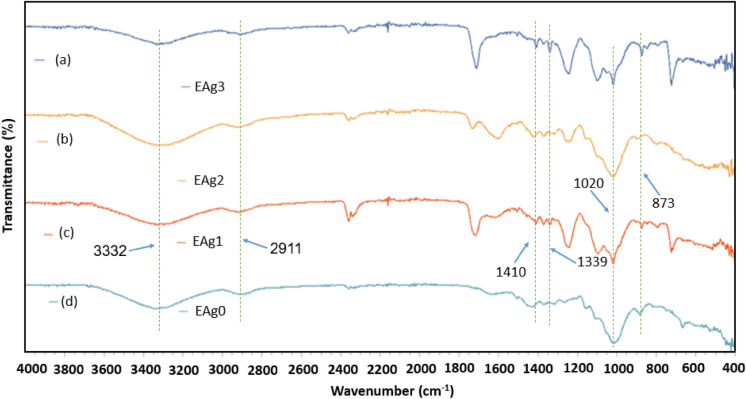
FTIR investigation of control and nanosilver-coated sisal/cotton interwoven fabrics.

The important characteristic bands of sisal/cotton interwoven fabrics and AgNP were explored using FTIR investigation (Fig. 7). Interestingly, no considerable shift was noticed before and after the nanosilver treatments on fabrics. The key characteristic bands of naturally derived sisal/cotton were found at 3332 cm^‒1^ (‒OH stretching), 2911 cm^‒1^ (C‒H stretching), and 1410 cm^‒1^ (C‒H wagging), 1339 cm^‒1^ for C‒H bending, 873 cm^‒1^ (C‒O stretching)^40,41^. The peaks around 1714 cm^‒1^ are related with the water absornbing molecules of cellulosic fibers^40^. The bands around 1020 cm^‒1^ are attributed to C‒O‒C stretching^42^. The treatment of sisal/cotton interwoven fabrics with nanosilver did not change the chemical structures of the fabrics as no new peaks appeared^43^. In some cases, broad peaks appear after nanosilver treament, which may be ascribed to the presence of European larch heartwood aquesous extracts in the system. However, the overall discussions demonstrate a successful bonding between the sisal/cotton interwoven fabrics and European larch heartwood mediated nanosilvers.

### Thermal property investigations of control and nanosilver-coated sisal/cotton interwoven fabrics

**Figure 8:**
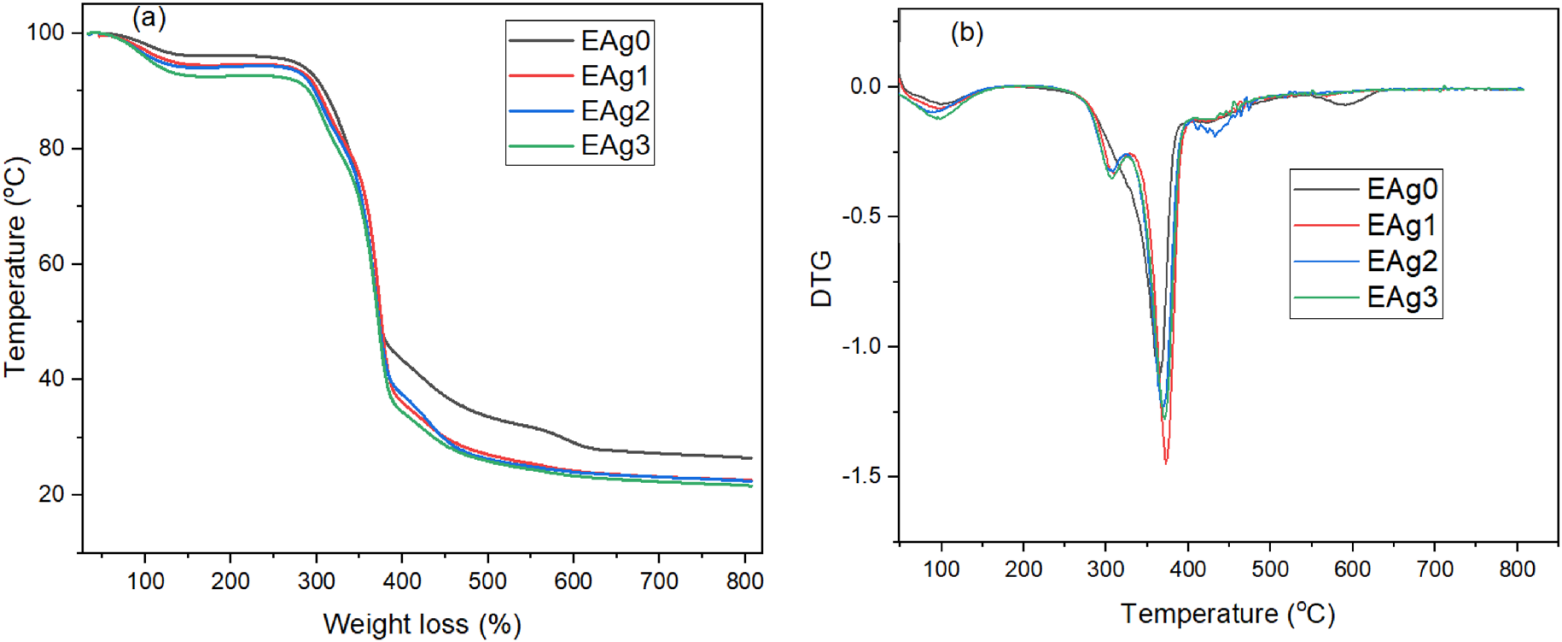
TGA and DTG analysis of control and nanosilver-coated sisal/cotton interwoven fabrics: (**a**) TGA and (**b**) DTG.

Neat sisal/cotton interwoven fabrics and European larch heartwood mediated AgNPs were subjected for TGA and DTG analysis under nitrogen atmosphere. The thermogram photographs are displayed in Fig. 8. As with other cellulosic materials in which cellulose is the key chemical element, the degradation on weight in sisal/cotton interwoven fabrics started at 90 to 100 °C with moisture evaporation^44^. The main weight loss occurred between 276 °C to 386 °C. This initial weight loss continued until 447 °C, demonstrating the degradation of cellulose^44^. Another significant weight loss occurred at around 600 °C^44^. The results confirm that European larch heartwood-mediated AgNP treatment slightly altered the thermal stability of sisal/interwoven fabrics when compared to the control, which concurs with the findings of a previous study^45^. The peaks around 304 °C are associated with the hemicellulose degradations of sisal/cotton materials^46^. Furthermore, the peaks around 364 °C for control sisal/cotton and around 375 °C for AgNP treated sisal/cotton indicate the cellulosic materials degradation from fibers^47^. The phenomena discussed above clearly demonstrate the behavior of cellulosic compounds from naturally derived sisal/cotton materials at certain temperatures.

## Conclusions

This study reported on the green synthesis of European larch heartwood mediated AgNPs deposited over sisal/cotton interwoven fabric with superior color characteristics and superior thermal stability. The key reason for the coloration is the LSPR properties of AgNPs in fabric surfaces during in situ synthesis protocol. The stated synthesis protocolol and methodology is scalable, inexpensive, eco-friendly, and controllable. The formation of regulated AgNPs were confirmed by iCP OES analysis of aqueous solution, EDX, and elemental mapping of developed sustainable products. The quantified values of AgNPs present in aqueous solutions were 16 mg/L, 323 mg/L, and 697 mg/L for samples 2, 3, and 4, respectively. Moreover, morphological observations of the products also display strong and uniform distributions of AgNPs over the sisal/cotton fabric surfaces. The perceived K/S values of the nanosilver treated samples were 2.74, 6.76, and 8.96 for samples 2, 3, and 4, respectively. Conversely, the control sample provided a K/S value of only 0.09. The reported products also displayed significant thermal stability when exposed to heat. The current study has outlined a novel and facile route, one that has not yet been reported and could prove to be a milestone for the coloration industries, offering them a new solution with which to replace traditional colorants. The regression analysis and ANOVA test also confirmed further the significance of increased AgNPs loading on the fabric samples. The overall performance characteristics of the developed materials-sustainable sisal/cotton interwoven fabrics as well as colorful and thermally stable textiles-exhibited an eco-friendly and novel pathway that industry can utilize.
